# The Role of Nrf2 Activity in Cancer Development and Progression

**DOI:** 10.3390/cancers11111755

**Published:** 2019-11-08

**Authors:** Alina-Andreea Zimta, Diana Cenariu, Alexandru Irimie, Lorand Magdo, Seyed Mohammad Nabavi, Atanas G. Atanasov, Ioana Berindan-Neagoe

**Affiliations:** 1 MEDFUTURE—Research Center for Advanced Medicine, Iuliu Hatieganu University of Medicine and Pharmacy, Marinescu 23 Street, 400337 Cluj-Napoca, Romania; zimta.alina.andreea@gmail.com (A.-A.Z.); diana.cenariu@umfcluj.ro (D.C.); 2 Department of Surgical Oncology and Gynecological Oncology, Iuliu Hatieganu University of Medicine and Pharmacy, 400015 Cluj-Napoca, Romania; airimie@umfcluj.ro; 3 Department of Surgery, The Oncology Institute “Prof. Dr. Ion Chiricuţa”, 400015 Cluj-Napoca, Romania; 4 Research Center for Functional Genomics, Biomedicine and Translational Medicine, Iuliu Hatieganu University of Medicine and Pharmacy, Marinescu 23 Street, 400337 Cluj-Napoca, Romania; lorand.magdo@gmail.com; 5 Applied Biotechnology Research Center, Baqiyatallah University of Medical Sciences, 14359-16471 Tehran, Iran; nabavi208@gmail.com; 6 Institute of Genetics and Animal Breeding of the Polish Academy of Sciences, 05-552 Jastrzebiec, Poland; atanas.atanasov@univie.ac.at; 7 Department of Pharmacognosy, University of Vienna, Althanstrasse 14, A-1090 Vienna, Austria; 8 Institute of Neurobiology, Bulgarian Academy of Sciences, 23 Acad. G. Bonchev str., 1113 Sofia, Bulgaria; 9 Department of Functional Genomics and Experimental Pathology, The Oncology Institute “Prof. Dr. Ion Chiricuta”, Republicii 34-36 Street, 400015 Cluj-Napoca, Romania

**Keywords:** Nrf2, Keap1, signaling pathway, oxidative stress, cancer, targeted genes

## Abstract

Nrf2 is a transcription factor that stimulates the expression of genes which have antioxidant response element-like sequences in their promoter. Nrf2 is a cellular protector, and this principle applies to both normal cells and malignant cells. While healthy cells are protected from DNA damage induced by reactive oxygen species, malignant cells are defended against chemo- or radiotherapy. Through our literature search, we found that Nrf2 activates several oncogenes unrelated to the antioxidant activity, such as Matrix metallopeptidase 9 (*MMP-9*), B-cell lymphoma 2 (*BCL-2*), B-cell lymphoma-extra large (*BCL-xL*), Tumour Necrosis Factor *α* (*TNF-α*), and Vascular endothelial growth factor A (*VEGF-A*). We also did a brief analysis of The Cancer Genome Atlas (TCGA) data of lung adenocarcinoma concerning the effects of radiation therapy and found that the therapy-induced Nrf2 activation is not universal. For instance, in the case of recurrent disease and radiotherapy, we observed that, for the majority of Nrf2-targeted genes, there is no change in expression level. This proves that the universal, axiomatic rationale that Nrf2 is activated as a response to chemo- and radiation therapy is wrong, and that each scenario should be carefully evaluated with the help of Nrf2-targeted genes. Moreover, there were nine genes involved in lipid peroxidation, which showed underexpression in the case of new radiation therapy: *ADH1A*, *ALDH3A1*, *ALDH3A2*, *ADH1B*, *GPX2*, *ADH1C*, *ALDH6A1*, *AKR1C3*, and *NQO1*. This may relate to the fact that, while some studies reported the co-activation of Nrf2 and other oncogenic signaling pathways such as Phosphoinositide 3-kinases (*PI3K*), mitogen-activated protein kinase (*MAPK*), and Notch1, other reported the inverse correlation between Nrf2 and the tumor-promoter Transcription Factor (TF), Nuclear factor kappa-light-chain-enhancer of activated B cells (*NF-κB*). Lastly, Nrf2 establishes its activity through interactions at multiple levels with various microRNAs. MiR-155, miR-144, miR-28, miR-365-1, miR-93, miR-153, miR-27a, miR-142, miR-29-b1, miR-340, and miR-34a, either through direct repression of Nrf2 messenger RNA (mRNA) in a Kelch-like ECH-associated protein 1 (Keap1)-independent manner or by enhancing the Keap1 cellular level, inhibit the Nrf2 activity. Keap1–Nrf2 interaction leads to the repression of miR-181c, which is involved in the Nuclear factor kappa light chain enhancer of activated B cells (NF-κB) signaling pathway. Nrf2’s role in cancer prevention, diagnosis, prognosis, and therapy is still in its infancy, and the future strategic planning of Nrf2-based oncological approaches should also consider the complex interaction between Nrf2 and its various activators and inhibitors.

## 1. Introduction

The process of tumor formation and dissemination of malignant cells is strictly regulated by the external exposure of cells to harmful or protective elements. The interplay between these factors is seen at the cellular level through the strict modulation of signaling pathways [1,2,3] for which the activation or inhibition can either sustain or impair malignant transformation of normal cells and enhanced aggressiveness of cancer cells. Kelch-like ECH-associated protein 1(Keap1)-Nuclear factor erythroid 2-related factor 2 (Nrf2) is the most studied signaling pathway of cellular defense against oxidative stress [4,5,6]. The *NFE2L2* gene encodes the Nrf2 protein [7], which is part of the “Cap ’n’ Collar” (CNC) family of transcription factors (TFs) [8].

When reactive oxygen species (ROS) level rises, Nrf2 disassociates from its inhibitor, Keap1, and enters into the nucleus. Keap1 belongs to the Kelch family of proteins (KLHL), and it binds to the Nrf2-ECH homology (Neh2) domain of Nrf2 in a region containing 69–84 amino acids flanked by the ETGE motif [9]. The ubiquitination of lysine in the Neh2 domain and consequent proteasomal degradation is mediated by Keap1, which represents the link between Nrf2 and the ubiquitination ligase Cullin-3 (Cul-3) [10]. Translational, post-translational, transcriptional, and epigenetic mechanisms also regulate Nrf2 distribution, as well as other protein partners. P62 is an adaptor protein which delivers ubiquitinated proteins to the autophagosome. This protein interacts with Keap1, thus increasing the intranuclear concentration of Nrf2 [11]. At the same time, p62 is a target gene for the Nrf2 transcription factor [12]. P21 is a positive regulator of Nrf2, via direct interaction, which stabilizes the Nrf2 protein [13]. The globular adiponectin (gAcrp) induces the production of the pro-inflammation Tumour Necrosis Factor *α* (TNF-α) and Interleukin 1 beta (IL-1β) in adipocyte-associated macrophage, but also raises the level of p62, a negative regulator of pro-inflammatory cytokines [14]. P62 binds to Keap1 and mediates its degradation [11,15].

Inside the nucleus, NRF2 forms a heterodimer with small V-Maf Avian Musculoaponeurotic Fibrosarcoma Oncogene MAF proteins (sMAF) and regulates the expression of genes that contain the antioxidant response elements (AREs) or the MAF recognition elements (MARE) in their promoter region [16,17]. Nrf2 controls an estimated number of 250 genes, across different species involved mainly in endogenous antioxidant protection and detoxification of reactive oxygen species (ROS) [18,19].The following genes are some of its most important targets: NAD(P)H dehydrogenase (quinone) 1 (*NQO1*), Glutathione S-transferase 1 (*GST*) [20], heme oxygenase-1 (*HMOX1*), glutamate–cysteine ligase (*GCL*), peroxiredoxins, and Glutathione (*GSH*) synthesis enzyme [21]. Mutation of Nrf2 or mutations of its negative regulator Keap1 can disrupt their interactions, which may account for overactivation of Nrf2 signaling [22].

Several authors already showed the defensive role of Nrf2 in various malignancies, neurodegenerative diseases, cardiovascular disorders, aging, inflammation, or photo-oxidative stress [23]. Moreover, multiplication and overgrowth of tumoral cells were shown to take place due to hyperactivation of Nrf2, which prevents cancer cells from undergoing apoptosis and cellular death, thereby rendering it resistant to chemotherapy or radiotherapy [24].

There are several types of cancers in which this mechanism was demonstrated, and tumors were proven to use Nrf2 as a self-protective mechanism [25].

In this review, we analyze the involvement of Nrf2 in cancer installment and progression focusing on the interactions this signaling pathway establishes with various kinds of regulators: proteins, microRNAs, and interactions with other signaling pathways and transcription factors. We also take a look at the The Cancer Genome Atlas (TCGA) data regarding the activity of Nrf2 in lung cancer and how it affects the cellular response to radiation therapy.

## 2. The Nrf2 Involvement in Cancer Development and Progression

**Figure 1:**
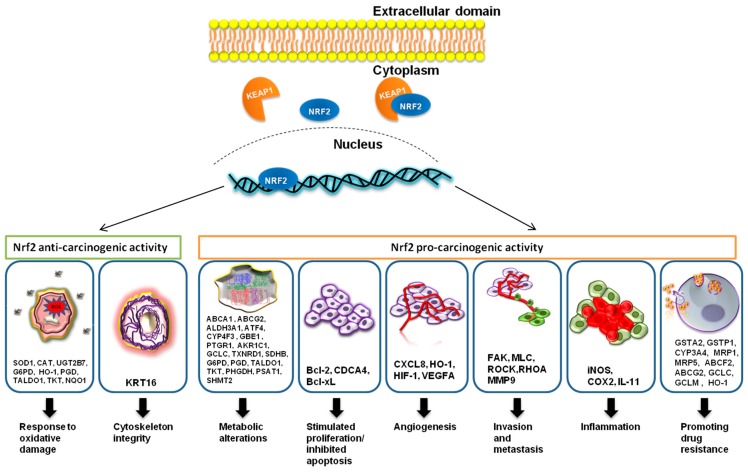
Nrf2, through its targeted genes, has an anti-carcinogenic role in the case of normal cells and a pro-carcinogenic effect in the case of transformed malignant cells. Nrf2 stimulates the expression of *SOD1, CAT, UGT2B7, G6PD, HO-1, PGD, TALDO1, TKT*, and *NQO1* genes, and it is involved in the protection of normal cells against intrinsic or extrinsic oxidative stress. Nrf2 also induces the expression of the *KRT16* gene, which helps in the maintenance of cytoskeleton integrity. In pre-cancerous lesions or cancerous cells, Nrf2 causes metabolic alterations such as anaerobic glycolysis, altered pentose phosphate pathway, and fatty-acid biosynthesis, among others, which induce the shift to anabolism with the help of the following targeted genes: *ABCA1, ABCG2, ALDH3A1, CYP4F3, GBE1, PTGR1, AKR1C1, GCLC, TXNRD1, SDHB, G6PD, PGD, TALDO1*, and *TKT*. Nrf2 activation also leads to the stimulated proliferation and inhibition of apoptosis, by enhancing the expression of the anti-apoptotic genes *BCL-2* and *BCL-XL* and the proliferation gene *CDCA4.* With the help of C-X-C Motif Chemokine Ligand 8 (*CXCL8*), Heme oxygenase 1 (*HO-1*), and Hypoxia-inducible factor 1 (*HIF-1*), Nrf2 was proven to induce angiogenesis in cancer. The overstimulation of Vascular endothelial growth factor A (*VEGFA*) could also be involved in cancer. Nrf2 can either stimulate or inhibit invasion and metastasis. The targeted genes involved in the stimulation are *FAK, MLC, ROCK, RHOA*, and *MMP9*. Nrf2, however, also downregulates the invasion-promoting gene, *Snail*. Nrf2 inhibits Nrf2-induced inflammation with the help of its targeted genes *iNOS, COX2*, and *IL-11*, while it also inhibits the expression of pro-inflammatory cytokines (not illustrated) *TNFα, IL-6, IL-8*, and *IL-1β*. The most important cancer-promoting action of Nrf2 is the promotion of chemoresistance through multiple mechanisms due to its targeted genes *GSTA2, GSTP1, CYP3A4, MRP1, ABCF2, ABCG2, GCLC, GCLM, MRP5*, and *HO-1*.

Nrf2 is a cytoprotective transcription factor which has both a positive effect and a negative effect on cancer [26,27]. Nrf2 protects both normal and cancerous cells from oxidative damage, meaning that it inhibits malignant transformation; however, once cancer is installed, it offers resistance to therapy [28] (Figure 1). Anti-cancer therapy acts as a selective force over the tumor cell population, and it stimulates the development of resistance. In the case of resistance to chemotherapy, these are fixing the drug-induced cell damage, creating a drug-suppressing environment, and avoiding intracellular drug exposure [29]. Nrf2 plays crucial roles in chemotherapy resistance by stimulating drug metabolism or drug efflux [24].

The different roles of Nrf2 in cancer progression were analyzed in a study from 2016, with Keap1 knockdown mice. The mice grew smaller urethane-induced tumors and had the following antioxidant genes upregulated: Catalase (Cat), Glutathione peroxidase 2 (Gpx2), Glutathione-S-transferase a4 (Gsta4), Ppargc1A and Glutathione reductase (Gsr). However, when these tumors were transplanted into nude mice, the tumors were more aggressive [30].

The reasons behind the overactivation of Nrf2 in cancer are DNA mutations, epigenetic changes, and modifications in the protein structure. In some cancer types, one or both Keap1 and Nrf2 are mutated, which does not allow a proper chemical interaction between the two [22,31,32,33]. For instance, Nrf2 is mutated in the Keap1-binding region in preneoplastic lesions in the liver [34].

In many cancers, the general methylation pattern of the DNA is changed, and the promoter region of Keap1 becomes hypermethylated, resulting in the decreased transcription of the Keap1 protein and the release of Nrf2 [31,35,36,37]. Another mechanism is the accumulation of fumarate and the succination of cysteine in Keap1 protein, which abolishes its sensitivity to oxidative stress [38,39], or the increased level of p21 [40] and p62 [41] proteins, which inhibit the interaction between Keap1 and Nrf2 [31,42].

In classical Hodgkin lymphomas, it was found that the increased expression of Nrf2 is associated with a limited form of the disease as opposed to the advanced forms [43]. In glioblastoma cells, Nrf2 overexpression leads to increased proliferation and oncogenic transformation [44].

Nrf2 is involved in maintaining cancer cell proliferation and invasion. In hepatocellular carcinoma, it was proven that the upregulation of the Nrf2 signaling pathway was correlated with the increased level of *MMP9* (matrix metallopeptidase 9) and *Bcl-xL* (B-cell lymphoma extra large). *MMP-9* is a metalloproteinase involved in cancer invasion through degradation of the basal membrane, and Bcl-xL is an anti-apoptotic factor [45]. In glioma cells, Nrf2 increased the expression level of *MMP9* [46]. The Nrf2 signaling pathway contributed to decreased apoptosis through the overstimulation of the anti-apoptotic protein, *Bcl-2*. There is an ARE region in the −3148 and −3140 reverse strands of the promoter region of *Bcl-2* [47].

In cervical cancer, the Nrf2 signaling pathway leads to increased proliferation and inhibits apoptosis [48]. In breast cancer, Nrf2 activation leads to the overexpression of Rho and the downstream proteins *FAK* (focal adhesion kinase 1), *MLC* (modulator of volume-regulated anion channel current 1), and *ROCK* (Rho-associated coiled-coil-containing protein kinase 1), while inhibiting the expression of estrogen-related receptor α (*ERR1*). Nrf2 also directly interacts with Breast cancer type 1 susceptibility protein (*BRCA1*), leading to increased stability of the BRCA protein. In the absence of *BRCA* expression, estrogen restores Nrf2 activation, causing decreased production of ROS in vitro and the protection of mammary gland cells [49]. The exogenous antioxidant, phospholipid hydroperoxide glutathione peroxidase (*PHGPx*), or pro-oxidant, 15-lipoxygenase (*15-LOX*), inhibited the expression of the vascular cell adhesion molecule (*VCAM*) via Nrf2 interaction in the promoter region of this gene [50].

There is a negative regulation between E-cadherin and the Nrf2 protein. E-cadherin impairs the nuclear localization of Nrf2, with the help of β-catenin. In hepatocellular carcinoma, however, E-cadherin is inhibited due to the action of Transforming Growth Factor Beta 1 (TGFβ) [51]. However, in lung fibrosis, it was reported that the Nrf2 protein inhibits epithelial-to-mesenchymal transition (EMT) by repressing the expression of *Snail* genes [52].

In bladder cancer tissue, the overexpression of *HO-1* was correlated with the increased expression of Nrf2, HIF-1α, HIF-2α, and VEGFA. In sera of bladder cancer patients, the pro-inflammatory cytokines IL-6 and IL-8 were also elevated, along with the pro-angiogenic factor VEGFA [53].

The TNF-α cytokine functions both as a promoter and as an inhibitor of the Nrf2 pathway. At average concentrations (2–5 ng/mL), TNF-α mediated the nuclear import of Nrf2 and the transcription initiation of its target genes, while, at high concentrations (> 10–50 ng/mL), it inhibited the Nrf2 pathway [54].

Hypoxic conditions are also regulators of the Nrf2 stimulation of transcription. In acute hypoxia, this TF binds less commonly in the enhancer or promoter region of *HMOX1*, than in the case of acute hypoxia [55].

The exposure of HepG2 cells to homocysteine results in the stabilization of Nrf2, and the transcription activation of glutamate cysteine ligase (*GCLc*). The stimulated expression of *GCLc* leads to the synthesis of glutathione, an important antioxidant, thus protecting the cancerous cells from the damage caused by high oxidative stress [56].

In mucoepidermoid carcinoma of the lung, HMOX1 overexpression was also associated with the inhibition of cell-cycle progression proteins Cyclin D1 (CCND1) and CCND2, and the stimulated transcription of the cell-cycle arrest proteins Gastrin (GAS) and Cyclin-dependent kinase inhibitor 1C (CDKN1C). The small interfering RNA (siRNA)-mediated silencing of HO-1 decreased the expression level of invasion promoters (*MMP-9*, *MMP-1*, and *MMP-12*), pro-inflammatory cytokines (*IL-6*, *IL-1β*, and *TNFα*), and the pro-angiogenic factor VEGFA [57]. An important finding is the fact that Nrf2 is overexpressed in lung cancer and breast cancer tissue, concomitant with the anti-inflammatory interleukin IL-11. This interleukin belongs to the IL-6 family, and it is associated with the inflammasome-induced epithelial cancers such as gastric and breast cancer [58]. Nrf2 antioxidant activity is involved in regulatory T cells (T regs) apoptosis, which counteracts the beneficial role of Programmed cell death 1 ligand 1 (PD-L1) antitumor therapy [59].

In laryngeal carcinoma tissue versus healthy adjacent tissue, it was found that Nrf2 is overexpressed and localized mainly in the nucleus, while Keap1, NQO1, and HO-1 were found mainly in the cytoplasm. There is a positive correlation between Nrf2, Keap1, NQO1, and HO-1 [60]. In the human hepatocarcinoma cell line, carbon monoxide (CO) enhanced the activity of Nrf2 and the *NQO1* gene [61].

An illustration of cancer-related genes modulated by Nrf2 is shown in Figure 1.

In cancer cells, it was proven that Nrf2 stimulates the multidrug-resistance-associated protein-1 (MRP1). Moreover, the *MRP1* gene has two ARE-binding regions (ARE1/2) in its promoter, which can explain the synchronous expression of Nrf2 and MRP1 [62]. Nrf2 also stimulates the expression of other genes which promote chemoresistance: ATP Binding Cassette Subfamily G Member 2 (*ABCG2*) [63], Glutathione S-Transferase Alpha 2 (*GSTA2*), Glutathione S-Transferase Pi 1 (*GSTP1*), Cytochrome P450 Family 3 Subfamily A Member 4 (*CYP3A4*), Heme Oxygenase 1 (*HO-1*), *MRP5* [64], ATP Binding Cassette Subfamily F Member 2 (*ABCF2*) [65], Glutamate-Cysteine Ligase Catalytic Subunit (*GCLC*), and Glutamate-Cysteine Ligase Modifier Subunit (*GCLM*) [66]. In colon cancer, Nrf2 is overexpressed, and, by elevating the expression of as P-glycoprotein (Pgp) and breast cancer resistance protein (BCRP), Nrf2 induces doxorubicin resistance by drug efflux [67]. Nrf2 is also involved in cell metabolism, which is one of the primary mechanisms for chemoresistance. It was proven that the overexpression of Nrf2 leads to changes in the cell metabolism; for instance, it changes anabolism [68], or it inhibits lipogenesis and simulates the β-oxidation of fatty acids [69]. Nrf2 regulates the genes involved in phosphate pentose pathways, such as Glucose-6-Phosphate Dehydrogenase (*G6PD*), Phosphogluconate Dehydrogenase (*PGD*), Transaldolase 1 (*TALDO1*), and Transketolase (*TKT*), and the synthesis of purines. Its activity involves the inhibition of Keap1 and the activation of the signaling pathway hosphatidylinositol-4,5-bisphosphate 3-kinase (PI3K)/RAC-alpha serine/threonine-protein kinase (Akt). Moreover, for metabolism reprogramming, Nrf2 also interacts and modulates the activity of other TFs such as p53, c-Myc, and HIF1α [70]. Nrf2 regulates genes involved in serine and glycine metabolism, such as Phosphoglycerate Dehydrogenase (*PHGDH*), Phosphoserine Aminotransferase 1(*PSAT1*), and Serine Hydroxymethyltransferase 2 (*SHMT2*) [71], or the glycolysis and the tricarboxylic acid cycle, by stimulating the expression of Abhydrolase Domain Containing 14B (*ABHD14B*), Acyl-CoA Thioesterase 13 (*ACOT13*), Aldo-Keto Reductase Family 1 Member C1 (*AKR1C1*), Aldehyde Dehydrogenase 3 Family Member B1 (*ALDH3B1*), Glutamate-Cysteine Ligase Catalytic Subunit(*GCLC*), Kynureninase (*KYNU*), LDL Receptor Related Protein 8 (*LRP8*), Nicotinamide Phosphoribosyltransferase (*NAMPT*), Prostaglandin E Synthase (*PTGES*), Prostaglandin Reductase 1 (*PTGR1*), Solute Carrier Family 27 Member 5 (*SLC27A5*), and Thioredoxin Reductase 1 (*TXNRD1*) [72]. Nrf2 targets the Transketolase (*TKT*) gene, which encodes for an enzyme involved in the pentose phosphate pathway (PPP) and protects cancerous cells from treatment-induced oxidative stress [73].

The Nrf2 signaling pathway is, in turn, repressed by multiple nuclear receptors. Retinoid X receptor alpha (RXRα) and Peroxisome proliferator-activated receptor gamma (PPARγ) bind to the retinoic acid response element in DNA or the peroxisome proliferation-activated receptors. Estrogen receptor alpha (ERα), Estrogen-related receptor β (ERRβ), and Glucocorticoid receptor (GR) bind in the promoter region of their targeted genes [74].

Nrf2 stability is increased through the binding of Inhibitor of apoptosis-stimulating protein of p53 (iASPP), a negative regulator of the p53 signaling pathway, which cooperates with Keap1 for Nrf2 binding [75]. Nrf2-mediated cell survival after oxidative stress is induced by the Eukaryotic Translation Initiation Factor 2 Alpha Kinase 3 (EIF2AK3, also known as PERK) protein [76].

The wild-type p53 interacts with the promoter of Nrf2, and it induces its suppression; however, in the case of cancer, the mutated p53 can no longer bind to the promoter region of Nrf2, and it leads to the stimulated transcription of this gene and cisplatin resistance [77]. *P53* is a gene with essential functions in the progression of various cancer types, such as breast cancer [78], colorectal cancer [79], and lung cancer [80].

On the other hand, lung cancer cells which have overexpression of the Nrf2/HO-1 axis have downregulated expression of IL-1β and metallo-proteinases [57]. The activity status of NRF2 reported for various malignancies is shown in Table 1.

## 3. The Interplay between Nrf2 and Other Signaling Pathways

The Keap1/Nrf2 pathway interacts with other signaling pathways, such as NF-κB, PI3K/Akt, Notch, MAPK, and Wnt Family Member 3AWNT-3A. These switch the tumor survival signal on and off as a result of Nrf2 activation or inhibition (see Table 2).

The mitogen-activated protein kinase kinase 1 (MAP2K1, alternative name MEK)/extracellular signal–regulated kinase (ERK) signaling pathway induces the nuclear accumulation of Nrf2. In human embryonic kidney cells, HEK-293, it was proven that the MEK/ERK signaling pathway interacts with Nrf2 through IQ Motif Containing GTPase Activating Protein 1 (IQGAP1) [106]. IQGAP1 functions as a tumor-promoting protein in breast cancer [107], glioma [108], and ovarian cancer [109].

The Nuclear factor kappa light chain enhancer of activated B cells (NF-κB) signaling pathway promotes the expression of the pro-inflammatory cytokines TNFα, IL-1, IL-6, and IL-8 [110], and its activation is involved in tumor promotion [111]. NF-κB is another signaling pathway with a dual role in cancer which induces DNA damage causing cell death; however, as cancer progresses, it establishes many cancer hallmarks, including resistance to chemotherapy [112]. Nrf2 counteracts the NF-κB action through multiple mechanisms: stimulating the antioxidant genes, impairing the NF-κB signaling pathway, and inhibiting the production of pro-inflammatory cytokines [113].

Inhibitor of nuclear factor kappa-B kinase subunit beta (IKKβ) is a member of the NF-κB signaling pathway that interacts with Keap1 and induces IKKβ ubiquitination. The Keap1 genomic locus is lost or mutated in cancer [114]. The p65 protein competes with the cAMP response element-binding protein (CREB)-binding protein (CBP) for binding to ARE and impairs binding of CBP to ARE. P65 also interacts with histone deacetylase 3 (HDAC3) and causes the histone hypoacetylation of ARE. P65 overexpression is induced by NF-κB signaling [115]. MAF bZIP transcription factor K (MafK) forms a complex with CBP and Nrf2 that stimulates the transcription of several genes. This protein, however, also mediates the recruitment of p65 and NF-κB to the promoters of IL-8 and TNFα [116].

The PI3K/Akt/mechanistic target of rapamycin kinase (mTOR) signaling pathway promotes cancer cell proliferation and survival [117], especially through its essential role in cell-cycle progression [118]. Nrf2 was associated with the stimulation of the platelet-derived growth factor A (PDGFA) by recruiting specific proteins to its promoter. It also activates the Akt/p21 pathway, which leads to cell-cycle progression [119].

Nrf2 also leads to the increased transcription of the proliferating cell nuclear antigen (*PCNA*) gene and the activation of the Notch signaling pathways, which augments the cellular level of NOTCH1 intracellular domain (*NICD1*) and Hes Family BHLH Transcription Factor 1 (*HES1*) genes, leading to enhanced proliferation of oral squamous cell carcinoma cells [120].

P38 MAP kinase (MAPK) activation leads to the turning on of Nrf2, which resulted in acquired resistance to temozolomide in glioma cells [121].

## 4. The microRNA Regulation of Nrf2 Signaling Pathway in Cancer

MicroRNAs (miRNAs) are a type of non-coding RNA with a length of 19–25 nt [131] which can repress the translation of mRNA into protein and can target multiple types of mRNAs [132]. In cancer, microRNAs can either be overexpressed and promote cancer progression or underexpressed and inhibit cancer progression [133,134], and they are important regulators of multidrug resistance [135]. All microRNAs that target not only Nrf2 but also Keap1 and other proteins involved in the cell response to oxidative stress are called redox miRs [43].

MiR-432 is an oncomiR involved in the cisplatin resistance of cancerous cells, and it binds to the coding region of the Keap1 transcript. This is followed by the inhibition of Keap1–Nrf2 interaction and the activation of Nrf2 [136].

In bronchial epithelial cells exposed to arsenic, the level of miR-155 increased, and Nrf2 translation was inhibited [137]. Nrf2 translation is also repressed by miR-144-3p in the peripheral blood and bone marrow of acute myeloid leukemia (AML) patients. MiR-144-3p is overexpressed in HL-60 cells, and it is involved in apoptosis resistance and cell viability promotion [138]. MiR-28 binds to the 3’ untranslated region (UTR) of Nrf2 and suppresses the mRNA ribosomal translation. This is a direct binding and does not impair the Nrf2–Keap1 interaction [139]. MiR-148b inhibits Nrf2 expression by down regulating *ERMPI* expression [140].

In breast cancer, the silencing of Nrf2 leads to the activation of the NF-κB signaling pathway and the overexpression of miR-181c. This miRNA inhibits the mitochondrial production of cytochrome c oxidase, the mitochondrial potential maintenance, and oxygen consumption [141].

Because Nrf2 is a transcription factor, it can also induce the transcription of several microRNAs by binding to the promoter region of the encoding DNA sequence. These targeted miRNAs include miR-193b, miR-365, miR-617, miR-592, miR-1207, miR-32, miR-200c, and miR-550 [142]. Nrf2 induces the transcription of HO-1, which in turn downregulates the activity of DiGeorge Syndrome Critical Region Gene 8 (DGCR8), and it is involved in miRNA biogenesis. The microRNAs regulated by Nrf2 can have a feedback loop response and regulate their transcription. MiR-27a, miR-142-5p, miR-144, and miR-153 downregulate Nrf2 production in a Keap1-dependent mechanism. MiR-21 is also a generally regarded oncomiR with key functions in inflammation, and, by being a downstream effector of the TGFβ-initiated pathway, it is also involved in the epithelial-to-mesenchymal transition. MiR-155 is involved in cytokine production in cancerous cells [143]. Nrf2 opposed the TGFβ/miR-21 activity in alcohol-treated lung fibroblasts [144].

In mucoepidermoid lung carcinoma (MEC), Nrf2 overexpression was correlated with HO overexpression, and it led to MMP12 and MMP9 downregulation. Nrf2 production was positively correlated with miR-181a, miR-193b, and miR-424 and negatively associated with miR-378 [57].

During the process of carcinogenesis, miR-365-1, miR-193b, miR-28, miR-93, miR-153, miR-27a, miR-142-5p, and miR-144 are downregulated. This underexpression leads to the upregulation of their target, Nrf2 mRNA, and the stimulated phosphorylation and activity of this transcription factor. Nrf2 overstimulation results in increased cell survival, sustained tumorigenesis, and enhanced tumor growth. This is followed by an increased miR-125b1 and decreased miR-29-b1 expression level, which offers chemoresistance. During chemotherapy, ROS levels are also increased, as well as miR-141 and miR-340 expression level, which suppress the phosphorylation of Nrf2. MiR-200a is decreased during carcinogenesis, which inhibits the formation of the Nrf2–Keap1–Cul3 complex [145]. MiR-200a was found to target Keap1 mRNA and, thus, release Nrf2 from the cytoplasm entrapment [146,147]. Nrf2 and miR-200a overexpression also promotes dendritic cell maturation [148].

In lung fibroblasts exposed to radiation, the BRCA1 level is increased, which elevates Nrf2 nuclear import, as well as decreasing the Keap1 level. Nrf2, once in the nucleus, promotes the transcription of miR-140, which results in impaired self-renewal ability, increased cell migration, and higher contractile capabilities [149].

An in vitro experiment proved that, in hepatocellular carcinoma, miR-141 is overexpressed, leading to Keap1 underexpression and, consequently, Nrf2 nuclear accumulation. This TF binds to the promoter region of the *HO-1* gene by increasing this gene product and triggers an elevated 5-fluorouracil resistance [97]. In the same cancer type, it was proven that miR-340 is downregulated, and its target Nrf2 is upregulated. Nrf2 overexpression confers cisplatin resistance to hepatocellular carcinoma cells through its antioxidant activity [150].

The feedback regulation between Nrf2 and its miRNA targets was also revealed in AML cells. Through Keap1 silencing, miR-125b was upregulated, and miR-29b was downregulated by Nrf2 in AML. It was further proven that the Nrf2 binds to the 5’ UTR DNA region of miR-125b and downstream of miR-29b. The altered expression of these two microRNAs results in leukemic cell survival after chemotherapy treatment [151].

In mouse keratinocytes, Nrf2 activation leads to the overstimulated transcription of miR-29a/b by Nrf2 bonding to the promoter region of the MiR-29ab1 gene. Nrf2 also leads to changes in epidermal desmosomes [152].

Metformin, an antidiabetic drug repositioned for cancer [153], induces Sirtuin 1 (*SIRT*) downregulation only in p53 wild-type cancer cells. This results in the upregulation of p53 and miR-34a, followed by a decreased level of Nrf2 and PPARγ transcriptional activity [154].

In human leukemic cells, Mitogen-activated protein kinase 7 (MAPK7, also known as ERK5) and Myocyte Enhancer Factor 2 (MEF2) are phosphorylated in response to oxidative stress. MEF2 enters the nucleus, where it stimulates the transcription of miR-23a. This microRNA represses the translation of Keap1 in the cytoplasm [155].

**Figure 2:**
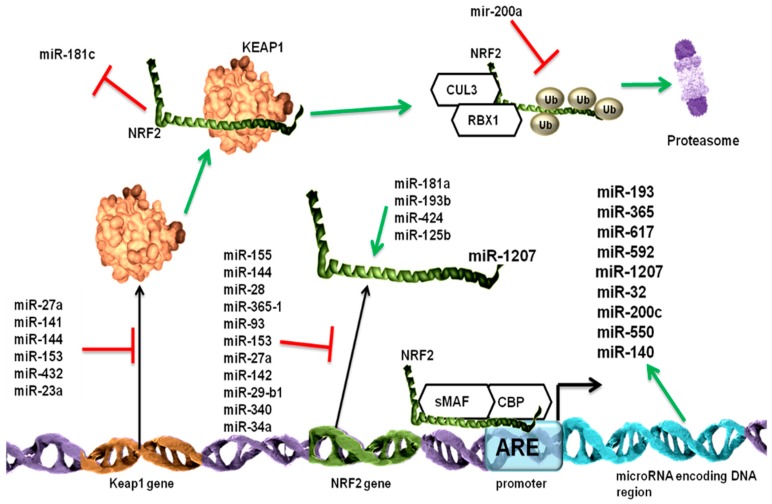
The Nrf2 pathway is regulated at multiple levels by different microRNAs. The Nrf2 pathway has two important players: the Nrf2 transcription factor and its inhibitor, the Kelch-like ECH-associated protein 1 (Keap1) protein. The Nrf2 interaction with Keap1 leads to the further binding of Cullin 3 (Cul3) and RING-box protein 1 (Rbx1) proteins and the ubiquitination of Nrf2 proteins, resulting in proteasomal degradation. If Nrf2 does not bind to Keap1, it binds to the antioxidant response element (ARE)-containing promoter regions, and, by forming a complex with small V-Maf Avian Musculoaponeurotic Fibrosarcoma Oncogene (sMaf) and cAMP response element-binding protein (CREB)-binding protein (CBP), it leads to the further activated transcription of its targeted genes. MicroRNAs suppress Keap1/Nrf2 signaling at multiple levels. MiR-27a, miR-141 miR-144, miR-153, miR-200a, miR-432, and miR-23a repress the translation of Keap1 messenger RNA (mRNA), thus allowing for Nrf2 activation. MiR-155, miR-144, miR-28, miR-365-1, miR-93, miR-153, miR-27a, miR-142, miR-29-b1, miR-340, and miR-34a, either through direct repression of Nrf2 mRNA in a Keap1-independent manner or by enhancing the Keap1 cellular level, inhibit Nrf2 activity. MiR-181a, miR-193b, miR-424, and miR-125b stimulate Nrf2 activation most probably by repressing its activators. The Keap–Nrf2 interaction leads to the repression of miR-181c, which is involved in the Nuclear factor kappa light chain enhancer of activated B cells (NF-κB) signaling pathway. MiR-200a inhibits the ubiquitination of Nrf2, thus protecting it from degradation. Nrf2, in addition to the classical targeted genes, can also stimulate the expression of the miRNA-encoding DNA region. The Keap1 protein structure was taken from the http://www.rcsb.org database [156,157] and the Nrf2 structure was taken from the http://www.cisreg.ca/tfe/structures/NFE2L2-4780-1jnmB.pdb.png online tool.

The multi level regulation of Keap1-Nrf2 signaling pathway by microRNAs is illustrated in Figure 2.

## 5. Examples of Nrf2 Application in Evaluating Therapy Response

Throughout our review, we analyzed the protective role of Nrf2 in the case of cancer, which leads to a weaker treatment response. In lung cancer, it was proven in vitro that A549 cells transfected with Nrf2siRNA were more prone to cell death as a response to radiotherapy than the control A549 cells [158]. At the same time, as Tian et al. pointed out, it should be considered that Nrf2 also protects healthy cells from radiation-induced inflammation and a rise in oxidative stress [159]. The radiotherapy action is mediated mainly by enhancing the intracellular oxidative stress. The cancer stem cells gain radioresistance by activating DNA repair mechanisms, diminishing ROS level with the help of stimulated glutathione biosynthesis, activating anti-apoptotic pathways, and decreasing the intratumoral hypoxia [160]. Ionizing radiation induces the overexpression of Nrf2-targeted genes and offers radioresistance [161]. This can constitute the basis for developing new therapeutic strategies in cancer that are based on nanoscale targeted delivery of cytotoxic drugs [162,163].

Our analysis of TCGA data is meant to be proof of concept, which we used to prove that gene expression analysis in regard to Nrf2signaling pathways should not be so heavily focused on the expression of Keap1–Nrf2 mRNA expression, but also on the expression of their targeted genes.

In order to observe the consequences following Nrf2 activation in lung cancer tissue exposed to radiation therapy, we extracted clinical data and RNA-sequencing (RNA-Seq) data of lung adenocarcinoma (LUAD) from TCGA, which included 173 samples for which there was information regarding radiation therapy status. The clinical data selected for our analysis included qualitative values describing whether the patients went through radiation therapy or not. From the RNA-Seq dataset, we looked only at the genes whose transcription is induced by Nrf2, resulting in a total of 117 genes (and gene variants) (the list was taken from Reference [164], and for every gene all gene variants were searched for in the database) (see File S1, Supplementary Materials). Based on radiation therapy status, the dataset was divided into patients who received initial radiation therapy or not those who received and new radiation therapy or not. Additional radiation therapy is applied in the case of LUAD recurrence or progression of disease after initial therapy. We compared patients going through radiation therapy and those who did not go through radiation therapy by applying the non-parametric Mann–Whitney test.

**Figure 3:**
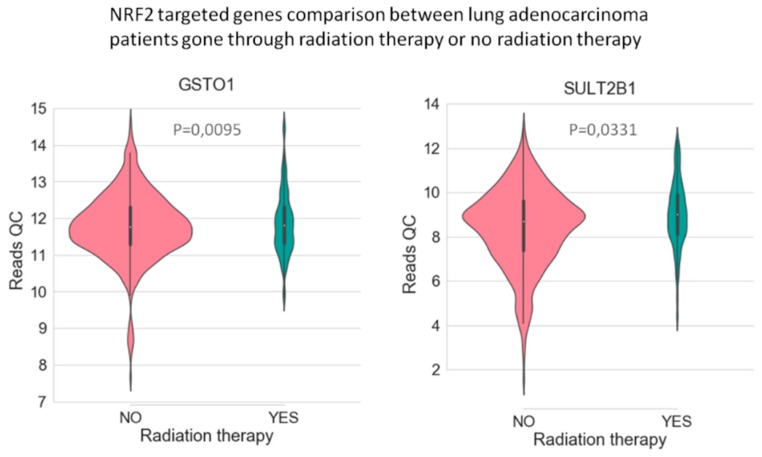
The Nrf2-targeted genes *GSTO1* and *SULT2B1* are upregulated in lung cancer tissue from patients who went through initial radiation therapy. The *p*-value was calculated with the help of the Mann–Whitney statistical test.

**Figure 4:**
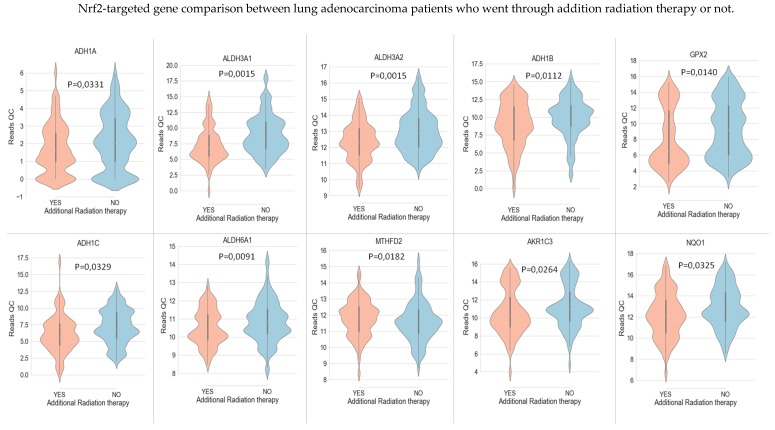
The Nrf2-targeted genes *MTHFD2* is upregulated in lung cancer tissue from patients who went through additional radiation therapy, while there are nine Nrf2-targeted genes which are downregulated in the case of additional radiation therapy: *ADH1A*, *ALDH3A1*, *ALDH3A2*, *ADH1B*, *GPX2*, *ADH1C*, *ALDH6A1*, *AKR1C3*, and *NQO1*. The *p*-value was calculated with the help of the Mann–Whitney statistical test.

In LUAD tissue samples from patients who initially received radiation therapy, we found only two genes, Glutathione S-Transferase Omega 1 and Sulfotransferase Family 2B Member 1 (from a total of 117 Nrf2-targeted genes), which were upregulated in the case of radiotherapy versus non-recipients of this kind of therapy (Figure 3). In LUAD tissue samples from patients who received initial radiation therapy, we found only one Nrf2targeted gene, *Methylenetetrahydrofolate Dehydrogenase (NADP+ Dependent) 2*, which was upregulated in the case of additional radiotherapy versus non-recipients of this kind of additional therapy. At the same time, nine genes were downregulated in the case of radiotherapy versus non-recipients of this kind of therapy (Figure 4). These genes were Alcohol Dehydrogenase 1A (*ADH1A*), Aldehyde Dehydrogenase 3 Family Member A1 (*ALDH3A1*), Aldehyde Dehydrogenase 3 Family Member A2 (*ALDH3A2*), Alcohol Dehydrogenase 1B (*ADH1B*), Glutathione Peroxidase 2 (*GPX2*), Alcohol Dehydrogenase 1C (*ADH1C*), Aldehyde Dehydrogenase 6 Family Member A1 (*ALDH6A1*), Aldo-Keto Reductase Family 1 Member C3 (*AKR1C3*), and NAD(P)H Quinone Dehydrogenase 1 (*NQO1*) (from a total of 117 Nrf2 targeted genes) All of these genes are involved in redox reactions.

**Figure 5:**
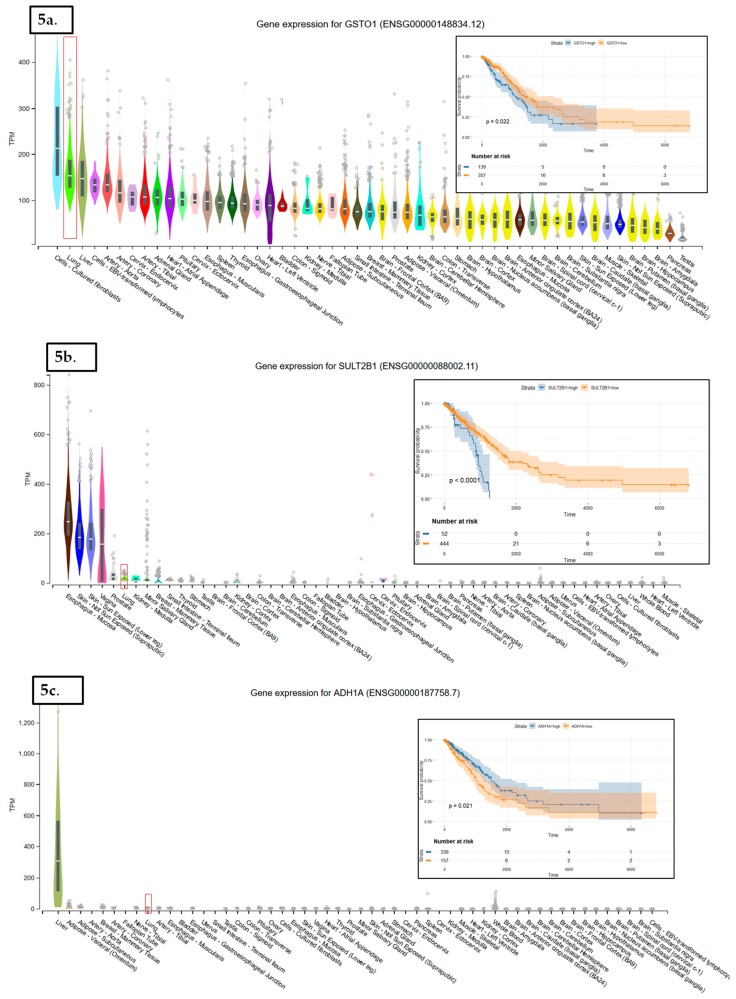

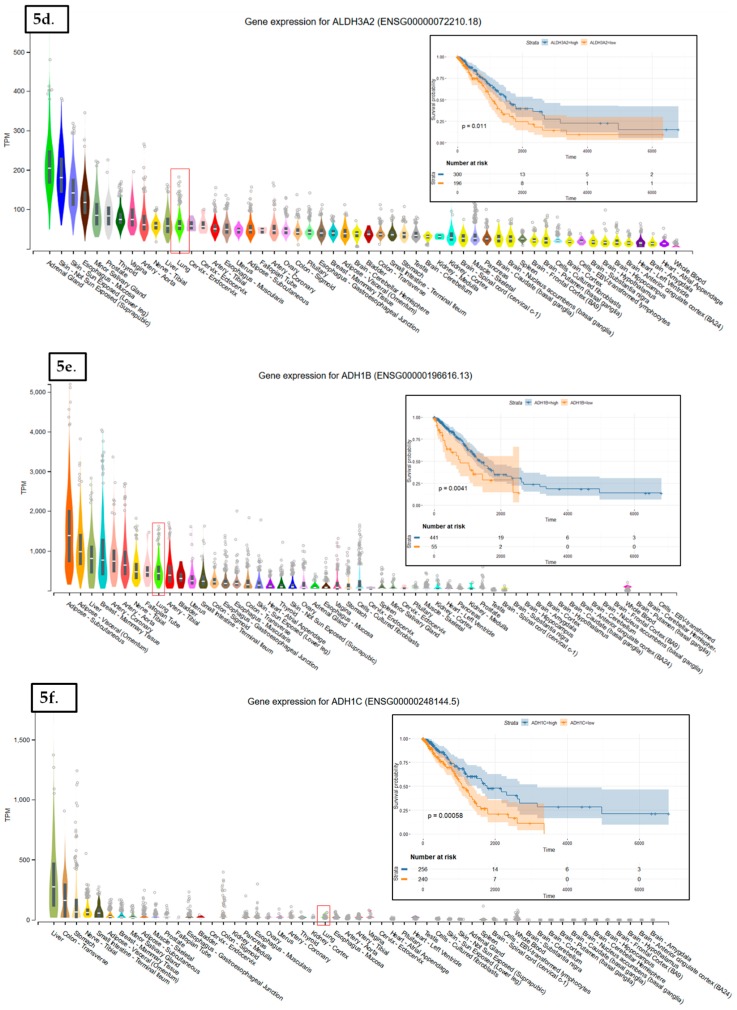

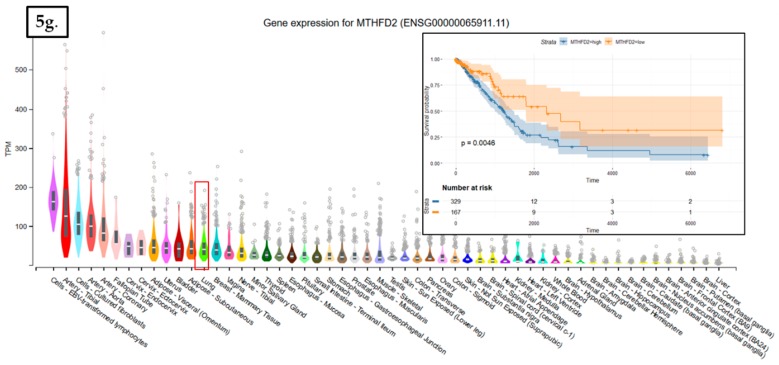
The tissue distribution and Kaplan–Meier (KM) plots of Nrf2-targeted genes affected by radiation therapy and whose expression affects the overall survival of lung cancer patients. These genes are *GSTO1* (**a**), *SULT2B1* (**b**), *ADH1A* (**c**), *ALDH3A2* (**d**), *ADH1B* (**e**), *ADH1C* (**f**), and *MTHFD2* (**g**). The expression of each gene in the lung normal tissue is marked by a red square. *GSTO1* (**a**), *SULT2B1* (**b**), *ADH1A* (**c**), *ADH1B* (**e**), and *ADH1C* (**f**) are downregulated in lung cancer versus normal tissue; *ALDH3A2* (**d**) and *MTHFD2* (**g**) are upregulated in lung cancer tissue versus normal adjacent tissue. On KM plots, groups with high expression are shown in blue, and groups with low expression are shown in red.

**Figure 6:**
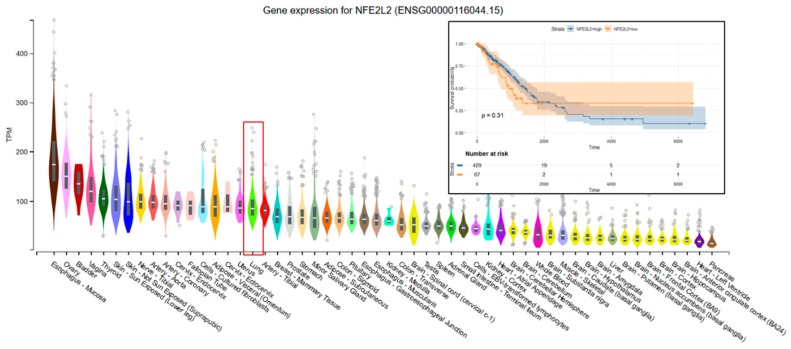
The tissue distribution and Kaplan–Meier plots of the Nrf2 gene (*NFE2L2*). The expression of each gene in the lung normal tissue is marked by a red square. On KM plots, groups with high expression are shown in blue, and groups with low expression are shown in red. The expression of the *NFE2L2* gene at the mRNA level has no significant effect on the overall survival of lung cancer patients.

The data used to look at the gene expression of each targeted gene affected by radiation therapy described in this manuscript were obtained from the GTEx Portal (https://gtexportal.org) on 31 October 2019. The survival analysis was done with the help of the RTCGA package and the maxstat (maximally selected rank statistics) package to determine the cutoff for each gene in R (Figure 5). From the total genes targeted by Nrf2 and affected by radiation therapy in lung cancer, we illustrated only seven genes with a statistically significant contribution to overall survival (log-rank, *p* < 0.05). These were Glutathione S-Transferase Omega 1 (*GSTO1*), Sulfotransferase Family 2B Member 1 (*SULT2B1*), *ADH1A*, *ALDH3A2*, *ADH1B*, *ADH1C*, and Methylenetetrahydrofolate Dehydrogenase (NADP+ Dependent) 2, Methenyltetrahydrofolate Cyclohydrolase (*MTHFD2*) (Figure 5). Nrf2 (*NFE2L2* gene) has no statistically significant influence in overall survival in lung cancer; however, the lung is the tissue with top *NFE2L2* expression (Figure 6). We also checked the expression of these genes in cancer tissue, especially lung cancer, with the help of the gepia online tool (http://gepia.cancer-pku.cn/index.html) [165], with a Log2FC cutoff of 1, and matched the TCGA normal and GTEx data. Glutathione S-Transferase Omega 1 (*GSTO1*), Sulfotransferase Family 2B Member 1 (*SULT2B1*), *ADH1A*, *ADH1B*, and *ADH1C* were downregulated in lung cancer versus normal tissue, while *ALDH3A2* and *MTHFD2* were upregulated in lung cancer tissue versus normal adjacent tissue.

**Figure 7:**
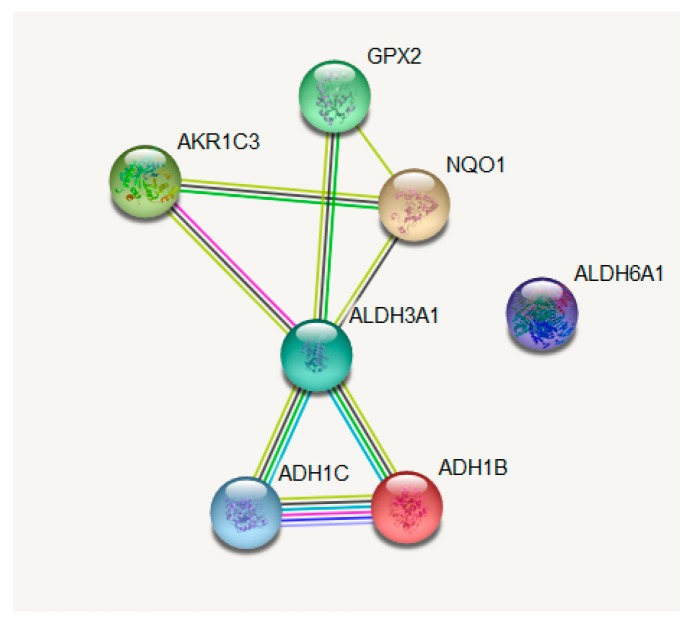
Interaction map of the Nrf2-targeted genes which show differential expression between lung adenocarcinoma (LUAD) patients who went through additional radiation therapy or not. The network was built with the help of the Gene String online tool (https://string-db.org/) [166].

The genes were then inserted in the Gene String online tool (https://string-db.org/), where the outliers were excluded from the central network (Figure 7); the function of these genes was also taken from the Gene String database (Table 3).

## 6. Conclusions

Nrf2 was suggested as a possible therapeutic target. Nrf2 implication in cancer remains controversial due to its protection of normal and cancerous cells. Nrf2 can be regarded as a simple response element to the signals initiated by an external factor or via its intersection with different regulatory mechanisms inside the cell. Nrf2 activation protects normal cells from malignant transformation, especially in the case of bronchial epithelial cells. At the DNA level, methylation in the Keap1 promoter, epigenetic silencing of its targeted genes, or mutations present in the binding sites of Keap1–Nrf2 take place during cancer development, but also under selective pressure of different therapies.

MicroRNAs can control the Nrf2 signaling pathway at different levels: repression of Keap1 by miR-27a, miR-141, miR-144, miR-153, miR-200a, miR-432, and miR-23a; repression of Nrf2 by miR-155, miR-144, miR-28, miR-365-1, miR-93, miR-153, miR-27a, miR-142, miR-29-b1, miR-340, and miR-34a; and the indirect activation of Nrf2 by miR-181a, miR-193b, miR-424, and miR-125b. Also, it is worth mentioning that the TCGA data revealed the fact that Nrf2 activation is not universal.

For instance, in the case of recurrent disease and radiotherapy, we observed that, for the majority of Nrf2-targeted genes, there is no change in expression level. This proves that the universal, axiomatic rationale that Nrf2 is activated as a response to chemo- and radiation therapy is wrong, and that each scenario should be carefully evaluated with the help of Nrf2-targeted genes. There were nine genes involved in lipid peroxidation, which showed underexpression in the case of additional radiation therapy. In addition to this, we consider that it makes more sense and it will have greater scientific value if future research on Nrf2 activation/inhibition in different scenarios is evaluated not through the evaluation of Keap1–Nrf2 mRNA expression, but through the mRNA expression of their targeted genes. It is also important to analyze the targeted genes at the post-transcriptional level, not the post-translational or protein levels, since there can be other inhibitory molecular mechanisms interfering in gene expression.

## Figures and Tables

**Table 1:** The Nrf2 status and effect in different cancer types.

Type of Cancer	Nrf2—Activated/Inhibited	Effect	Reference
Bladder cancer	Activated	Cisplatin resistance	[81]
Breast cancer	Activated	Increased resistance to taxol and anchorage-independent growth	[82]
		Increase as the disease progresses, leads to poor prognostic (p62)	[83]
		Upregulation leads to aromatase-induced drug resistance	[84]
		Decreased sensitivity to photodynamic therapy (PDT)	[85]
		Cell proliferation and migration	[86]
		Enhanced glycolysis	[87]
Cervical cancer	Activated	Drug resistance	[88]
Colon cancer	Inhibited	Epidermal Growth Factor (EGF) increases the expression of Nrf2	[89]
	Activated	5-Fluorouracil resistance Oxaliplatin resistance	[90,91]
Gastric cancer	Activated	Stimulates cancer progression in wild-type p53 cancers Worse prognosis	[92,93,94]
Glioblastoma	Activated	Radioresistance	[95]
Glioma	Activated	Stimulates the cancer stem-cell phenotype	[96]
Hepatocellular carcinoma	Activated	5-Fluorouracil resistance	[97]
Lung cancer	Activated	Supplies the substrates for glutathione and nucleotide production	[71]
		Mutated p53 does not inhibit the promoter region of *Nrf2* gene	[77]
		Increased chemoresistance	[62,98]
Multiple myeloma	Activated	Regulates endoplasmic reticulum (ER)-stress-associated apoptosis	[99]
Pancreatic cancer	Activated	Malignant transformation	[100]
		Chemoresistance	[101]
		Nrf2 activation counteracts the transforming growth factor beta (TGFβ) inhibition of premalignant lesions in pancreatic cancer	[102]
Prostate cancer	Inhibited	Promoter was found to be hypermethylated	[103]
		Lowered the reactive oxygen species (ROS) in prostate cancer (PCa) cells and sensitized PCa cells to radiation	[104]
Ovarian cancer	Activated	Resistance to methyl pyropheophorbide a-mediated photodynamic therapy	[105]

**Table 2:** The interaction between Nrf2 and other signaling pathways.

Pathways Interacting with Nrf2	Inhibition/Stimulation Activity	Effect	Reference
Nuclear factor kappa light chain enhancer of activated B cells (NF-κB)	↓ Nrf2 ↑ NF-κB	Enhanced Inhibitor of nuclear factor kappa-B kinase subunit beta (IKKβ) activity, degradation of nuclear factor of kappa light polypeptide gene enhancer in B-cells inhibitor, alpha (IκBα)	[122]
	↑ Nrf2 ↑ Glutathione (GSH) ↓ p65		[123]
	↓ p65 ↑ Nrf2–CREB-binding protein (CBP) complex association	Increased Nrf2 binding to CBP (cAMP response element-binding protein (CREB)-binding protein)	[115]
	Rac Family Small GTPase 1 (RAC1) → Nrf2 levels ↑	Protective mechanism against the inflammation consequences	[124]
	Lipopolysaccharides (LPS) → Rac Family Small GTPase 1(RAC)1 → Nrf2-mediated Heme Oxygenase 1 (HO-1) ↑		
	↑ p62 ↑ Nrf2	Modulation of antioxidant and inflammatory activities	[15]
	↑ p62 ↑ Tumour Necrosis Factor α (TNFα)		
Phosphoinositide 3-kinases (PI3K)/RAC-alpha serine/threonine-protein kinase (Akt)	↓ Nrf2 ↓ Nicotinamide adenine dinucleotide phosphate (NAD(P)H)	Proliferation of cancer cell lines with constitutive Nrf2 accumulation	[68,125,126]
	↑ PI3K ↑ Nrf2	*Pten* deletion → Nrf2 function →antioxidant response elements (ARE)-driven gene expression by Nrf2	
	↑ PI3K/Akt ↑ Nrf2	Modulation of the metabolic activity in the liver	
	↑ PI3K/Akt ↑ Nrf2	PI3K activation enhances the nuclear availability of Nrf2	
Notch 1	↑ Nrf2 ↑ Notch1 and Hes1 messenger RNA (mRNA)	Promotes proliferation, migration, invasion, cell cycle, and colony formation of Oral Squamous Cell Carcinoma (OSCC) cells	[120]
	↓ Nrf2 ↓ Notch1 and Hes1 mRNA		
Wingless-Type MMTV Integration Site Family, Member 3A (WNT-3A)	↑ Nrf2 ↑ WNT-3A ↑ HO-1 ↑ β-Catenin ↓ Axin1	Modulates hepatocyte metabolism	[127]
Kirsten Rat Sarcoma Viral Oncogene Homolog (K-Ras), v-Raf murine sarcoma viral oncogene homolog B (B-Raf), Myc	↑ Nrf2 activity ↑ K-Ras	Initiates pancreatic and lung tumorigenesis and proliferation	[128]
Proteosomal ubiquination	↑ p62 ↑ Nrf2	Kelch-like ECH-associated protein 1 (Keap1) inactivated; alterations of selective autophagy	[129]
B-Raf Proto-Oncogene, Serine/Threonine Kinase (MAPK)	↑ p38 ↑ c-Jun N-terminal kinases (JNK) ↑ Nrf2	MEQ (mequindox) induced redox imbalance damage in the mouse liver	[130]

**Table 3:** List of Nrf2-targeted genes and their function. The list was constructed with the help of the Gene String online tool (https://string-db.org/) [166].

Name	Biological Function
*ADH1B*	Belongs to the zinc-containing alcohol dehydrogenase family.
*NQO1*	The enzyme is apparently involved in detoxification pathways, as well as in the biosynthetic vitamin K-dependent gamma-carboxylation of glutamate residues in prothrombin synthesis.
*AKR1C3*	Catalyzes the conversion of aldehydes and ketones to alcohols. Catalyzes the reduction of prostaglandin (PG) D2, PGH2, and phenanthrenequinone (PQ) and the oxidation of 9-alpha, 11-beta-PGF2 to PGD2. Preferentially transforms androstenedione (4-dione) to testosterone.
*GPX2*	Could play a major role in protecting mammals from the toxicity of ingested organic hydroperoxides. *tert*-Butyl hydroperoxide, cumene hydroperoxide, and linoleic acid hydroperoxide can act as acceptors.
*ALDH3A1*	Plays a major role in the detoxification of alcohol-derived acetaldehyde. Involved in the metabolism of corticosteroids, biogenic amines, neurotransmitters, and lipid peroxidation.
*ADH1C*	Alcohol dehydrogenase.
*ALDH6A1*	Plays a role in valine and pyrimidine metabolism. Binds fatty acyl-CoA; aldehyde dehydrogenases.

## Supplementary Materials

The following are available online at https://www.mdpi.com/2072-6694/11/11/1755/s1, Table S1: Full list of Nrf2 targets included in our analysis.
