# Supplementary material for: The Role of Nrf2 Activity in Cancer Development and Progression
Source: Cancers (Basel). 2019 Nov 8;11(11):1755. doi: 10.3390/cancers11111755 (PMC6896028; doi:10.3390/cancers11111755)
Supplement: Supplementary file 1 [file cancers-11-01755-s001.pdf]

cancers
Supplementary Materials

Table S1. Full list of Nrf2 targets included in our analysis.

ENSEMBL_GENE_ID	Gene Name
ENSG00000196139	aldo-keto reductase family 1 member C3(AKR1C3)
ENSG00000138109	cytochrome P450 family 2 subfamily C member 9(CYP2C9)
ENSG00000242515	UDP glucuronosyltransferase family 1 member A10(UGT1A10)
ENSG00000103222	ATP binding cassette subfamily C member 1(ABCC1)
ENSG00000143149	aldehyde dehydrogenase 9 family member A1(ALDH9A1)
ENSG00000170899	glutathione S-transferase alpha 4(GSTA4)
ENSG00000106258	cytochrome P450 family 3 subfamily A member 5(CYP3A5)
ENSG00000231852	cytochrome P450 family 21 subfamily A member 2(CYP21A2)
ENSG00000140465	cytochrome P450 family 1 subfamily A member 1(CYP1A1)
ENSG00000114770	ATP binding cassette subfamily C member 5(ABCC5)
ENSG00000224586	glutathione peroxidase 5(GPX5)
ENSG00000196616	alcohol dehydrogenase 1B (class I), beta polypeptide(ADH1B)
ENSG00000106605	biliverdin reductase A(BLVRA)
ENSG00000167996	ferritin heavy chain 1(FTH1)
ENSG00000240224	UDP glucuronosyltransferase family 1 member A5(UGT1A5)
ENSG00000276950	glutathione S-transferase theta pseudogene 1(GSTTP1)
ENSG00000087086	ferritin light chain(FTL)
ENSG00000165092	aldehyde dehydrogenase 1 family member A1(ALDH1A1)
ENSG00000165841	cytochrome P450 family 2 subfamily C member 19(CYP2C19)
ENSG00000117448	aldo-keto reductase family 1 member A1(AKR1A1)
ENSG00000171428	N-acetyltransferase 1(NAT1)
ENSG00000138061	cytochrome P450 family 1 subfamily B member 1(CYP1B1)
ENSG00000109193	sulfotransferase family 1E member 1(SULT1E1)
ENSG00000001084	glutamate-cysteine ligase catalytic subunit(GCLC)
ENSG00000140505	cytochrome P450 family 1 subfamily A member 2(CYP1A2)
ENSG00000241119	UDP glucuronosyltransferase family 1 member A9(UGT1A9)
ENSG00000160868	cytochrome P450 family 3 subfamily A member 4(CYP3A4)
ENSG00000125257	ATP binding cassette subfamily C member 4(ABCC4)
ENSG00000167468	glutathione peroxidase 4(GPX4)
ENSG00000111275	aldehyde dehydrogenase 2 family (mitochondrial)(ALDH2)
ENSG00000186350	retinoid X receptor alpha(RXRA)
ENSG00000196344	alcohol dehydrogenase 7 (class IV), mu or sigma polypeptide (ADH7)
ENSG00000105227	periaxin(PRX)
ENSG00000142657	phosphogluconate dehydrogenase(PGD)
ENSG00000099984	glutathione S-transferase theta 2 (gene/pseudogene)(GSTT2)
ENSG00000053371	aldo-keto reductase family 7 member A2(AKR7A2)
ENSG00000136810	thioredoxin(TXN)
ENSG00000197448	glutathione S-transferase kappa 1(GSTK1)
ENSG00000134184	glutathione S-transferase mu 1(GSTM1)
ENSG00000121270	ATP binding cassette subfamily C member 11(ABCC11)
ENSG00000105398	sulfotransferase family 2A member 1(SULT2A1)
ENSG00000245848	CCAAT/enhancer binding protein alpha(CEBPA)

ENSG00000138413	isocitrate dehydrogenase (NADP(+)) 1, cytosolic(IDH1)
ENSG00000085563	ATP binding cassette subfamily B member 1(ABCB1)
ENSG00000198704	glutathione peroxidase 6(GPX6)
ENSG00000128918	aldehyde dehydrogenase 1 family member A2(ALDH1A2)
ENSG00000187758	alcohol dehydrogenase 1A (class I), alpha polypeptide(ADH1A)
ENSG00000119711	aldehyde dehydrogenase 6 family member A1(ALDH6A1)
ENSG00000172831	carboxylesterase 2(CES2)
ENSG00000066926	ferrochelatase(FECH)
ENSG00000091262	ATP binding cassette subfamily C member 6(ABCC6)
ENSG00000211445	glutathione peroxidase 3(GPX3)
ENSG00000278540	acetyl-CoA carboxylase alpha(ACACA)
ENSG00000243955	glutathione S-transferase alpha 1(GSTA1)
ENSG00000124574	ATP binding cassette subfamily C member 10(ABCC10)
ENSG00000128059	phosphoribosyl pyrophosphate amidotransferase(PPAT)
ENSG00000099194	stearoyl-CoA desaturase(SCD)
ENSG00000104687	glutathione-disulfide reductase(GSR)
ENSG00000151632	aldo-keto reductase family 1 member C2(AKR1C2)
ENSG00000023839	ATP binding cassette subfamily C member 2(ABCC2)
ENSG00000168765	glutathione S-transferase mu 4(GSTM4)
ENSG00000148795	cytochrome P450 family 17 subfamily A member 1(CYP17A1)
ENSG00000100577	glutathione S-transferase zeta 1(GSTZ1)
ENSG00000160211	glucose-6-phosphate dehydrogenase(G6PD)
ENSG00000197894	alcohol dehydrogenase 5 (class III), chi polypeptide(ADH5)
ENSG00000198610	aldo-keto reductase family 1 member C4(AKR1C4)
ENSG00000137869	cytochrome P450 family 19 subfamily A member 1(CYP19A1)
ENSG00000159228	carbonyl reductase 1(CBR1)
ENSG00000143819	epoxide hydrolase 1(EPHX1)
ENSG00000108602	aldehyde dehydrogenase 3 family member A1(ALDH3A1)
ENSG00000132170	peroxisome proliferator activated receptor gamma(PPARG)
ENSG00000271303	sulfiredoxin 1(SRXN1)
ENSG00000213366	glutathione S-transferase mu 2(GSTM2)
ENSG00000006071	ATP binding cassette subfamily C member 8(ABCC8)
ENSG00000131473	ATP citrate lyase(ACLY)
ENSG00000187134	aldo-keto reductase family 1 member C1(AKR1C1)
ENSG00000165568	aldo-keto reductase family 1 member E2(AKR1E2)
ENSG00000106546	aryl hydrocarbon receptor(AHR)
ENSG00000084207	glutathione S-transferase pi 1(GSTP1)
ENSG00000169710	fatty acid synthase(FASN)
ENSG00000072210	aldehyde dehydrogenase 3 family member A2(ALDH3A2)
ENSG00000244122	UDP glucuronosyltransferase family 1 member A7(UGT1A7)
ENSG00000233276	glutathione peroxidase 1(GPX1)
ENSG00000176153	glutathione peroxidase 2(GPX2)
ENSG00000164904	aldehyde dehydrogenase 7 family member A1(ALDH7A1)
ENSG00000065833	malic enzyme 1(ME1)
ENSG00000100292	heme oxygenase 1(HMOX1)
ENSG00000174156	glutathione S-transferase alpha 3(GSTA3)
ENSG00000163931	transketolase(TKT)

ENSG00000248144	alcohol dehydrogenase 1C (class I), gamma polypeptide(ADH1C)
ENSG00000088002	sulfotransferase family 2B member 1(SULT2B1)
ENSG00000130649	cytochrome P450 family 2 subfamily E member 1(CYP2E1)
ENSG00000116157	glutathione peroxidase 7(GPX7)
ENSG00000181019	NAD(P)H quinone dehydrogenase 1(NQO1)
ENSG00000100197	cytochrome P450 family 2 subfamily D member 6(CYP2D6)
ENSG00000255974	cytochrome P450 family 2 subfamily A member 6(CYP2A6)
ENSG00000198431	thioredoxin reductase 1(TXNRD1)
ENSG00000159423	aldehyde dehydrogenase 4 family member A1(ALDH4A1)
ENSG00000134201	glutathione S-transferase mu 5(GSTM5)
ENSG00000134538	solute carrier organic anion transporter family member 1B1 (SLCO1B1)
ENSG00000142168	superoxide dismutase 1, soluble(SOD1)
ENSG00000242366	UDP glucuronosyltransferase family 1 member A8(UGT1A8)
ENSG00000134202	glutathione S-transferase mu 3(GSTM3)
ENSG00000241635	UDP glucuronosyltransferase family 1 member A1(UGT1A1)
ENSG00000164294	glutathione peroxidase 8 (putative)(GPX8)
ENSG00000243135	UDP glucuronosyltransferase family 1 member A3(UGT1A3)
ENSG00000108846	ATP binding cassette subfamily C member 3(ABCC3)
ENSG00000177156	transaldolase 1(TALDO1)
ENSG00000261052	sulfotransferase family 1A member 3(SULT1A3)
ENSG00000148834	glutathione S-transferase omega 1(GSTO1)
ENSG00000156006	N-acetyltransferase 2(NAT2)
ENSG00000065911	methylenetetrahydrofolate dehydrogenase (NADP+ dependent) 2, methenyltetrahydrofolate cyclohydrolase(MTHFD2)
ENSG00000244067	glutathione S-transferase alpha 2(GSTA2)
ENSG00000138780	glutathione S-transferase C-terminal domain containing(GSTCD)
ENSG00000198848	carboxylesterase 1(CES1)
ENSG00000197408	cytochrome P450 family 2 subfamily B member 6(CYP2B6)
ENSG00000023909	glutamate-cysteine ligase modifier subunit(GCLM)
